# UV-B induced fibrillization of crystallin protein mixtures

**DOI:** 10.1371/journal.pone.0177991

**Published:** 2017-05-25

**Authors:** Sibel Cetinel, Valentyna Semenchenko, Jae-Young Cho, Mehdi Ghaffari Sharaf, Karim F. Damji, Larry D. Unsworth, Carlo Montemagno

**Affiliations:** 1 Department of Chemical and Materials Engineering, University of Alberta, Edmonton, AB, Canada; 2 Ingenuity Lab., University of Alberta, Edmonton, AB, Canada; 3 National Institute of Nanotechnology (NINT), 11421, Saskatchewan Drive NW, Edmonton, AB, Canada; 4 Department of Ophthalmology and Visual Sciences, University of Alberta, Edmonton, AB, Canada; Tsinghua University School of Life Sciences, CHINA

## Abstract

Environmental factors, mainly oxidative stress and exposure to sunlight, induce the oxidation, cross-linking, cleavage, and deamination of crystallin proteins, resulting in their aggregation and, ultimately, cataract formation. Various denaturants have been used to initiate the aggregation of crystallin proteins *in vitro*. All of these regimens, however, are obviously far from replicating conditions that exist *in vivo* that lead to cataract formation. In fact, it is our supposition that only UV-B radiation may mimic the observed *in vivo* cause of crystallin alteration leading to cataract formation. This means of inducing cataract formation may provide the most appropriate *in vitro* platform for in-depth study of the fundamental cataractous fibril properties and allow for testing of possible treatment strategies. Herein, we showed that cataractous fibrils can be formed using UV-B radiation from α:β:γ crystallin protein mixtures. Characterization of the properties of formed aggregates confirmed the development of amyloid-like fibrils, which are in cross-β-pattern and possibly in anti-parallel β-sheet arrangement. Furthermore, we were also able to confirm that the presence of the molecular chaperone, α-crystallin, was able to inhibit fibril formation, as observed for ‘naturally’ occurring fibrils. Finally, the time-dependent fibrillation profile was found to be similar to the gradual formation of age-related nuclear cataracts. This data provided evidence for the initiation of fibril formation from physiologically relevant crystallin mixtures using UV-B radiation, and that the formed fibrils had several traits similar to that expected from cataracts developing *in vivo*.

## Introduction

Cataracts are a leading cause of blindness worldwide and are characterized by opacification of the ocular lens. The only available current treatment for cataract is the surgical removal of the lens and replacement with a synthetic one. Alternative treatments would be enormously valuable, particularly for third-world countries due to the relatively excessive cost of the surgery and limited access to trained surgeons. Development of alternative therapeutic options is significantly hindered by the fact that an *in vitro* model of cataractous aggregates that mimics the naturally forming aggregates is not available for testing drugs, understanding aggregation initiation, etc. Considering cataract as a protein aggregation disease and applying a strategy similar to that utilized with neurodegenerative disorders, it’s possible to elucidate the fibrillation mechanism *in vitro* and further utilize these fibrils as a platform for drug testing. In this regard, research focusing on *in vitro* cataractous fibril formation from the aggregation of a variety of crystallin proteins has been intensely investigated under various denaturing conditions: heat, denaturant chemicals, pH shifts and UV-radiation [1–4].

Ocular lens transparency is maintained by multiple factors, including the presence of a high concentration of crystallin proteins and the level of chaperone content within the lens tissue [5]. Lens epithelial cells surrounding the lens capsule maintain the transport to the lens and continue to differentiate into fiber cells with a decline with age. The decreased α-crystallin expressions in lens epithelial cells could be associated with cataractogenesis [6–8]. Moreover, due to the fact that the fiber cells within the adult lens have lost their ability to express new proteins [9], any damage to crystallin proteins within the lens accumulates over time, and this accumulation ultimately causes a loss in protein solubility, leading to the formation of cataractous fibrils [5].

A wide variety of protein modifications have been identified in cataractous tissues: deamination, oxidation, racemization, truncation, phosphorylation and backbone cleavage [10, 11]. These modifications arise by the natural pathways of ageing (oxidative stress) [12] and exposure to ultraviolet light [13]. Even though, various denaturing conditions have been shown to initiate crystallin aggregation *in vitro*, the sensitivity of different crystallin classes varies depending on the denaturant. For instance, α-crystallin is not affected by heat inactivation as much as it is affected by UV-photodamage [14]. Moreover it’s shown that α-crystallin can enhance its chaperone activity against photodamage after partially unfolding its quaternary structure by pre-incubation at higher temperatures up to 60^°^C [15]. On the other hand β-crystallins are more resistant to UV-induced aggregation than are γ-crystallins [16–18]. More importantly, the different denaturant sensitivities of crystallins designate the pathway of aggregation and in return result in aggregates with divergent structures. For instance, at microscopic scale, it is possible to observe the formation of granular structures under heat denaturation versus fibrillar assembly under acid-induction of γ-crystallin [4]. When compared to using acid to induce aggregates, UV-B induced aggregation results in covalent structure alterations such as cross-linking, polypeptide cleavage, and side chain damage in γ-crystallin [19]. In light of this, it can be said that all crystallin aggregates will structurally differ from each other depending on the denaturant conditions and therefore it will be impossible to suggest either a generalized pathway for aggregation or a common solution for their reverse aggregation.

In order to build a platform to study the characterization and development of cataracts and to investigate alternative therapies for treating cataracts, it is crucial to have a system that forms cataractous fibrils under similar conditions as experienced *in vivo*. Among all applicable denaturant conditions, UV radiation may be the most relevant to disease initiation and progression. The effect of exposure to UV radiation in sunlight has already been associated with the formation of age-related cataracts [12, 20]. Also, it has been shown that UV induced aggregation of recombinant crystallins exhibit the same properties as proteins isolated from *in vivo* cataracts [21]. Within the UV spectrum, UVA (400–315 nm) lights that can penetrate deeper to the lens tissue are found to generate less damage to the ocular tissues compared to high-energy UVB (315–280 nm) lights, which are mostly absorbed by atmospheric ozone, upper eyelids and aqueous humor [22–27]. This effect can be associated with the presence of aromatic amino acid residues in crystallin proteins typically absorb in the wavelength range of UVB radiation [25, 28]. In general, photo-oxidation of proteins occurs due to the UV absorption by chromophore groups, which are tryptophan, tyrosine, phenylalanine, histidine and cysteine residues. Among them tryptophan residues are the most significant ones that absorb and filter most of the UV radiation within the range of 240–310 nm [29, 30]. However, long-term photo-oxidation results with their conversions -tryptophan to *N*-formylkynurenine and kynurenine, methionine to sulphoxide and cysteine to cysteine-, which introduces additional groups to the protein and therefore affect their hydrophobicity, stability and unfolding dynamics [31, 32]. Besides, the backbone cleavages (fragmentation of the protein) and cross-linking between altered histidine residues and lysine, cysteine or other histidine residues emanate. Collectively these alterations are found to result with high molecular weight, aggregated protein structures [33]. The effect of UV damage on lens tissue is prevented by the presence of UV filters. However, as a result of aging process, the concentration of UV filters in the lens decreases over time and the UV damage can be observed and lead to cataract formation [33].

So far, the aggregation profiles and aggregate structures of different crystallin classes have been studied under ultraviolet radiation and provide valuable information for individual proteins [34–37]. However lens tissue is composed of three different crystallin classes (namely α, β and γ-crystallins). Accordingly, aggregates formed within the lens are composed of various crystallin proteins. For this reason, we investigated the UV-B induced aggregation of recombinant α:β:γ crystallin mixtures in different ratios. Although it is evident that all three crystalline classes are found in *in vivo* aggregates, this situation is rarely studied in as systematic a manner as presented herein. Our results confirmed the development of amyloid-like fibrils, which were prone to protection by α-crystallin, as it should be in the natural eye. Thus, we believe, that mixture can serve as a platform for studying cataract formation and for testing the efficiency of drug candidates.

## Materials and methods

### Expression and purification of Human crystallin proteins

Protein sequences for the human crystallin αB (UniProtKB accession number P02511), human crystallin βB2 (UniProtKB accession number P43320) and human crystallin γD (UniProtKB accession number P07320) were converted into DNA sequences and codon optimization was applied for the protein expression in *Escherichia coli* cells. The human crystallin genes were inserted into a pET15b vector between the *NdeI* and *EcoRI* restriction sites. Enterokinase cleavage site (Asp-Asp-Asp-Asp-Lys) was coded into the N- terminal part of the each gene. The correct sequence, insertion and orientation of the crystallin constructs were verified by DNA sequencing.

Recombinant proteins were expressed in *E*. *coli BL21(DE3)* host cells and protein expression was induced with 1mM IPTG for 18 hr at 37°C. Both recombinant human crystallin proteins were purified with the same manner using metal affinity chromatography (IMAC) with Ni-NTA column and enterokinase digestion was performed for the removal of N-terminal His-tag. (Protocol A in S1 File).

Recombinant proteins were visualised with SDS-PAGE before and after His-tag removal (Protocol B and Fig A in S1 File). In Gel Protein Identification analyses were performed at Alberta Proteomics and Mass Spectrometry Facility—University of Alberta (Fig B in S1 File).

### UV-B induced fibrillization

Both individual and four different molar ratios of α, β and γ crystalline (α:β:γ as 0:2:1, 1:2:1, 5:2:1 and 10:2:1) proteins were prepared at 3mg/ml concentration in Phosphate buffer (10mM Sodium Phosphate, 200mM NaCl, pH 7.5). UV-B induced fibrillization was carried out by exposing proteins to UV-B (302 nm) at room temperature for 10 hours. The samples were incubated in sealed polypropylene tubes with 80% UV transmission. The temperature during the UV-B incubation was stable at 37^°^C till the end of the experiment. The initial control samples were incubated at either 37^°^C incubator or room temperature (RT) with no UV radiation during the course of the experiment. Since both of the control samples (room temperature or 37^°^C) resulted with the same color appearance and the same degree of aggregates, room temperature control used for the rest of the experiments. The samples collected in different time points (0.5, 1. 2, 5 and 10 hours) were analyzed with SDS-PAGE, ThT assay and TEM.

### Thioflavin T (ThT) assay

The presence of amyloid-like fibril structures in crystallin fibrils was confirmed with the increased fluorescent intensity in ThT assay. The assay was performed in Corning^®^ 96-well clear bottom black polystyrene fluorescence micro-plates by using 2μl protein (3mg/ml) diluted in 200 μl of 5 μM ThT solution (50 mM glycine-NaOH buffer, pH 9.0). The spectra was acquired by using a FlexStation^®^3 Multimode Microplate Reader (Molecular Devices, LLC.) at excitation wavelength of 440 nm and emission scanning wavelengths from 450–550 nm. Each sample was analyzed with three replicates and the average values were calculated. One-way ANOVA with post­hoc Tukey HSD Test was applied for statistical analysis.

### Circular dichroism (CD) analysis

CD spectra of proteins (0.05 mg/ml in 100 μM PBS) were obtained with a Jasco J-810 CD/ORD spectropolarimeter (Jasco Co., Tokyo, Japan) by using 1.0 mm cuvette. The spectrum derived represents an average of three scans with a smoothing factor of 9. The secondary structure predictions were acquired with DichroWeb [38, 39] by using CDSSTR [40–42] algorithm. The data fits with an NRMSD value >0.1 were considered as quality fits.

### Transmission electron microscopy (TEM) analysis

The fibril formation with different ratios of recombinant Human crystallin proteins αB, βB2 and γD were visualized by TEM (Hitachi H-9500, Hitachi Ltd.). Before applying the samples, carbon film on square mesh copper was glow discharged for 25 sec to increase hydrophilicity. 20μl of 3mg/ml protein (fibril) was applied to film for 10 seconds. The excess sample was blotted with filter paper and washed 3 times with 20 μl of buffer for 10 sec each. Then the samples were stained with 20 μl of 2% (w/v) uranyl acetate solution for 10 sec. This solution was blotted off and the grid was left in vacuum oven for 30 mins.

TEM images were analysed with ImageJ (Version 2.0) software. Particle analysis was performed on at least three images per sample. Fibrillar structures bigger than 5 nm in radius were counted. To start with, the background of the image has been subtracted with a balling roll tool of 50 pixels. Than, the image was adjusted to threshold where the aggregate structures have been identified. Finally the areas of particles have been analyzed with 0–1.0 circularity and the ones bigger than 25 nm^2^ have been counted.

### X-ray diffraction (XRD) measurements

The UV-B treated crystallin protein mixtures along with the control proteins were analyzed with X-ray diffraction to determine the presence of fibrils and understand the supramolecular arrangement of β-sheet structure. For the measurements, 5μl protein solution (15 mg/ml) was deposit on the glass slide and air-dried. The dried samples were collected to create a small clump and placed in the air to avoid the unnecessary peaks from substrate. A D8/Discover X-ray diffractometer (Bruker, Co.) with CuKa radiation (40kV, 40mA) was used to determine the X-ray diffraction patterns. 2 Theta ranges of each sample were from 0 to 26.5 degree with a step size of 0.01 degree. The beam stopper was used to reduce the beam intensity at the low angle (<2 degree). The collected data was analyzed by EVA^tm^ software including data smoothing (smooth factor at 1.5).

### Chaperone activity measurements

Lysozyme aggregation assay with slight modification was used to measure chaperone activity of α-crystallin [43]. Simply, the aggregation of 10μM lysozyme was initiated by 1mM TCEP (Tris-2-carboxyethyl phosphine) in PBS buffer and the turbidity of the reaction mixture was measured at 30°C for 1 hour in the absence and presence of α-crystallin (or crystallin mixtures such as 5:2:1 and 10:2:1). The lysozyme:α-crystallin molar ratio is kept 1:1 at all sample conditions. The alterations in α-crystallin activity due to UV-B exposure was tested by using UV-B treated samples. The turbidity in terms of absorbance change due to the aggregation of lysozyme was followed at 400 nm and recorded by using a FlexStation^®^3 Multimode Microplate Reader (Molecular Devices, LLC.).

## Results and discussion

### UV-B induced fibrillization of crystallin proteins

The lens tissue has the highest protein concentration in the body consisting of 35–45% of its weight [44]. Crystallin proteins constitute more than 90% of the total protein content of human lens tissue. The three major crystallin protein groups, namely, α, β and γ are found in a diversified ratio to each other in normal lens tissue [45]. Based on this work, we employed a solution ratio of 1:2:1 for α:β:γ, which is similar to the normal lens tissue protein distribution. In order to determine if UV-induced fibrillation was affected by α-crystallin content four different ratios of α, β and γ crystallin (α:β:γ as 0:2:1, 1:2:1, 5:2:1 and 10:2:1) at 3 mg/ml concentration, were studied. Briefly, all protein solutions were incubated under UV-B light up to 10 hours. UV−B radiation was generated through a transilluminator (Syngene, Ltd. GMV20) equipped with 6, 8Watt BL312 lamps with the output of 302 nm. The total UV intensity on the surface was ~50W/m^2^ and the irradiation was applied at a distance of 30 cm. It has already been determined that a 1 hour incubation under these conditions can mimic the 1h per day direct exposure to sea-level solar radiation for 3 years [46], yielding the equivalent of 30 years of 1 hour per day exposure to solar radiation.

**Fig 1:**
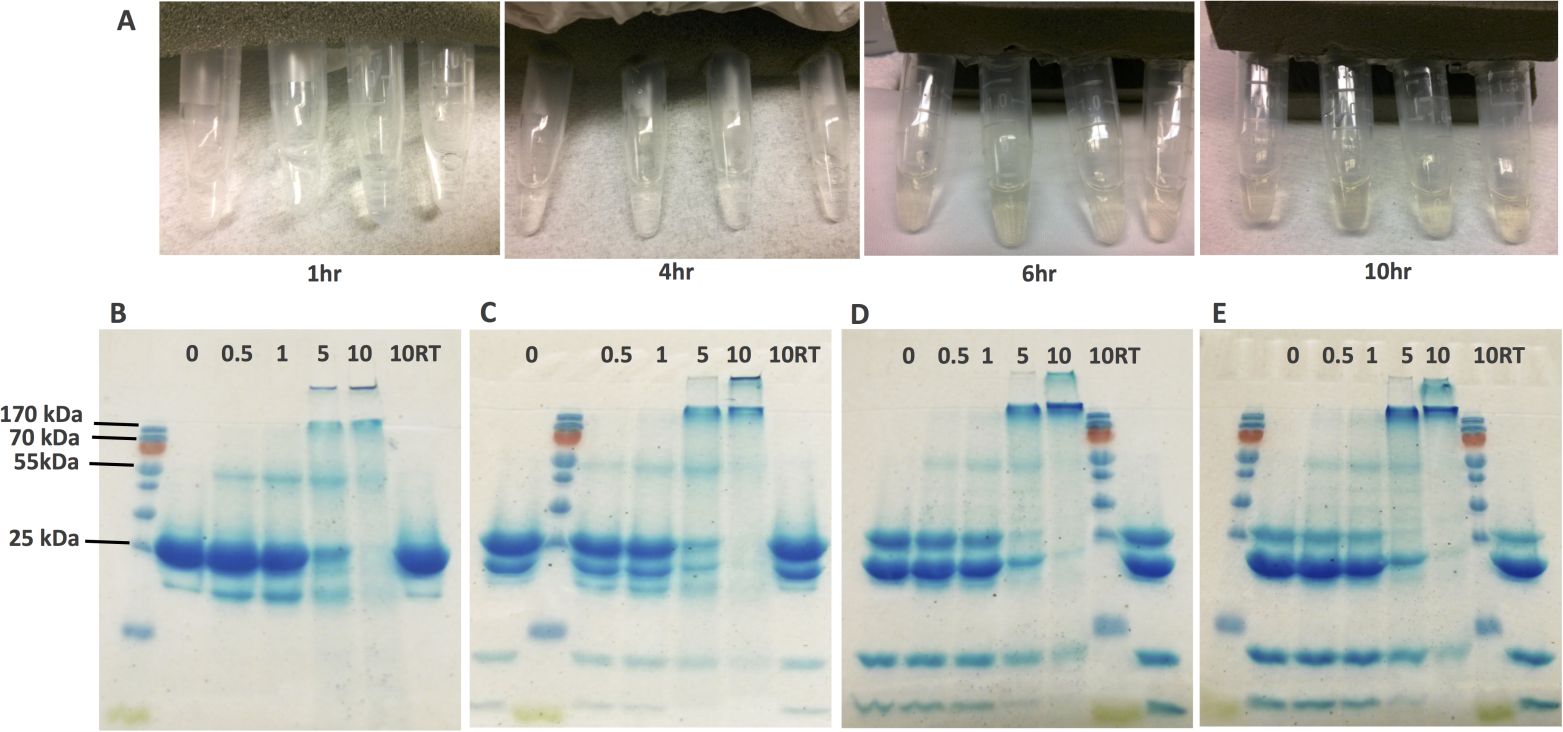
**(A)** The visual observation of different crystalline protein ratios for α:β:γ as 0:2:1, 1:2:1, 5:2:1 and 10:2:1 (tubes from left to right in both pictures) at different time points of UV-B radiation. SDS-PAGE of **(B)** 0:2:1, **(C)** 1:2:1, **(D)** 5:2:1 and **(E)** 10:2:1 ratio crystalline proteins. Upper lanes indicate the time points of UV exposure in hours (0, 0.5, 1, 5 and 10) and 10RT indicates 10 hours room temperature incubation (control sample).

The visual observation of the samples at different time points (1, 4, 6 and 10 hours) indicated a color change from transparent to a pale yellow color with respect to UV-B exposure period (Fig 1A), which is in agreement with previous studies suggesting the formation of aggregates [34]. That color change is also similar to the change seen in human cataracts with nuclear sclerosis [34]. On the other hand, the absorbance in visible range can be suggested to be consistent with photo-oxidative modification of aromatic side chains in the proteins [47].

SDS-PAGE gels in Fig 1 show the UV-treated crystallin mixtures of 0:2:1, 1:2:1, 5:2:1 and 10:2:1 ratios at different time points of exposure. Low molecular weight bands are visible in the gel images of 1:2:1, 5:2:1 and 10:2:1 ratios (Fig 1C–1E) at both time-0 and control samples (10RT- no UV exposure). Those low molecular bands can be traced back to the αβ-crystallin gel images in Fig A (in S1 File) and could be a result of non-mature expression of α-crystallin (since they are still observed after His-tag removal) or some *E*.*coli* proteins captured by α-crystallin as a result of its chaperon activity.

SDS-PAGE analysis of the samples revealed the formation of high molecular weight (HMW) aggregates during the course of the incubation (Fig 1B to 1E). For all the ratios, the dimer formation (the bands slightly lower than 55kDa) was observed in the first 30 min, which increased in concentration at the end of the first hour. While dimers were still observable by the end of the 5^th^ hour, HMW aggregates became dominant as can be seen as the bands higher than 170kDa. At the end of the 10^th^ hour, HMW aggregates became too large to enter the stacking gel. Meanwhile, the control samples, which were not subjected to UV-B light did not show a similar aggregation profile, indicating either the absence of HMW aggregates or the presence of reversible aggregates (in denaturing agents). Similar to previous results related to development of age-related nuclear cataracts, where it was observed that gradual changes in color occurred with time, our data shows (Fig 1) that the intensity of the HMW aggregates also increased with UV-B exposure time (Also see S1 Fig) [45]. Thus, it’s possible to conclude that UV-B radiation of crystallin proteins is an effective way of studying age-related cataract.

From these data, it seems apparent that UV-B radiation of these proteins formed aggregates that were unable to be solubilized using SDS and β-mercaptoethanol, suggesting UV-B radiation may induce irreversible changes to the protein structure [34]. It has been shown recently that dityrosine adducts might be the source of covalent linkages between proteins, as opposed to disulfide bridge, as they are known to be products of UV-B irradiation of proteins [47].

The depletion of monomeric proteins (20.1 kDa– α-crystallin, 23.4 kDa—β-crystallin and 20.7 kDa– γ-crystallin bands) has been observed during the 10 hours incubation period. After the 10 hr incubation very weak bands for monomeric proteins were still present, however it was not possible to identify the particular crystallin protein that was still in monomeric form. That said, the vast majority of proteins in the sample were incorporated into the newly formed HMW aggregates.

### Secondary structure alteration due to UV-B treatment

Cataractous aggregates can be present either as amorphous or amyloid-like fibrils [48]. Amyloid-fibrils are composed of well-organized cross-β-sheet structures, which are generated by protein association leading to fibril development. Crystallin proteins have been shown to form amyloid-fiber like aggregates both under denaturing conditions *in vitro* and during the aging process in human eyes [1, 2]. These fibrillar structures are comprised of a cross-β-sheet structure, where β-sheets are stacked perpendicular to the axis of the fibril [2]. In order to determine if these UV-induced aggregates were comprised of β-sheet secondary structures the ThT assay was employed. ThT incorporation to the β-sheet motifs result with increased fluorescence intensity and is accepted as a gold-standard assay for quantifying the presence of fibrils [49].

**Fig 2:**
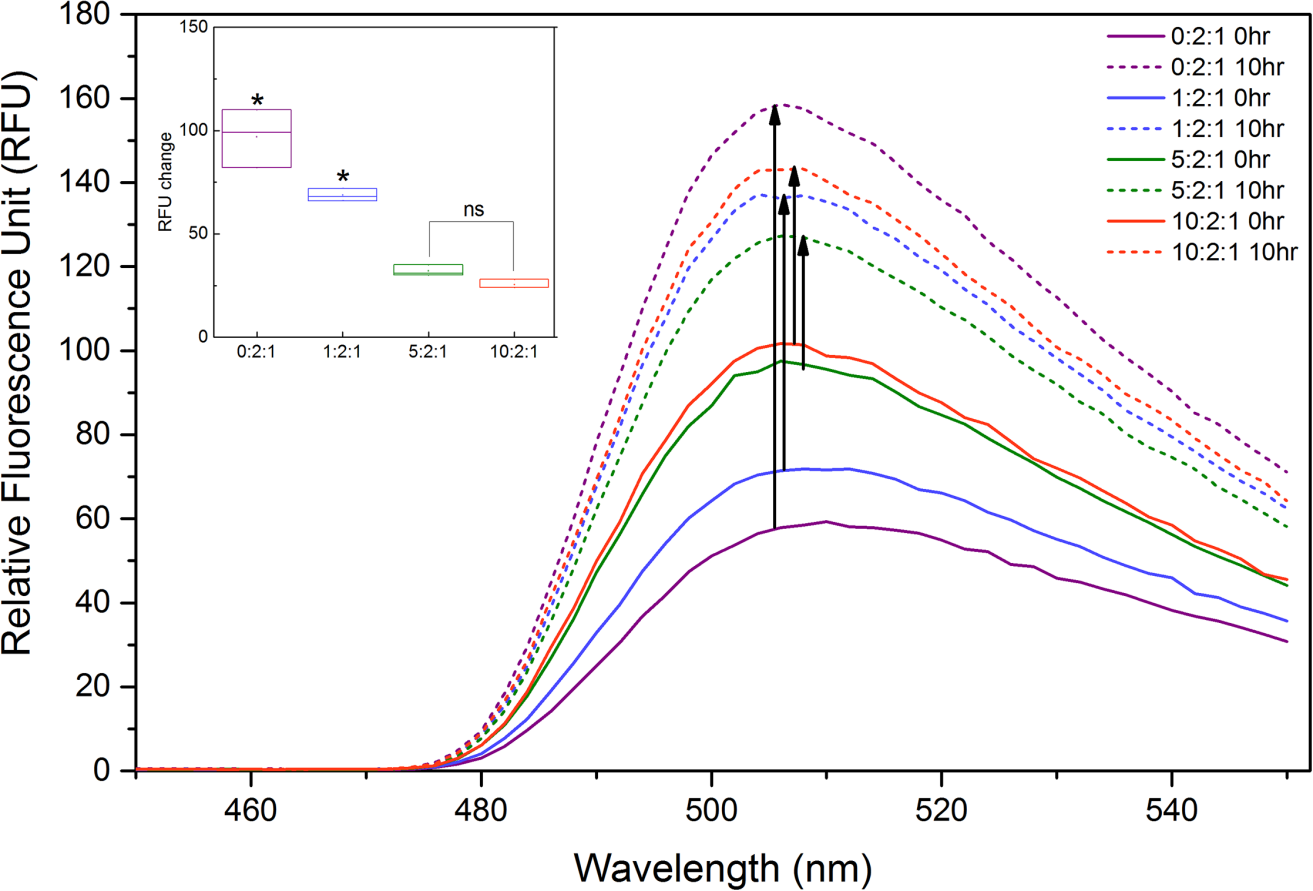
ThT fluorescence emission spectra of crystallin proteins in 0:2:1, 1:2:1, 5:2:1 and 10:2:1 ratios incubated at room temperature for 10 hours (solid lines) and exposed to UV-B radiation for 10 hours (dotted lines). **Inset graph** represents the relative intensity difference between UV-B treated and untreated samples. The data represents the average of 3 independent experiments and error bars represent SD. *: *P*<0.01, ns: Not Significant.

Since the β-sheet secondary structure dominates in all three native crystallin proteins [50, 51], fluorescence intensity for the UV-B untreated/control samples was recorded for a baseline comparison. Due to the different proportions of β-sheets in each crystallin protein [52], ThT signal observed for UV-untreated samples showed different intensities with respect to each other (Fig 2). However, the increase in fluorescent intensity in each sample as a function of UV-B treatment (Fig 2; Inset graph) clearly revealed the amyloid-like fibril formation. Among the studied ratios, 0:2:1 showed the highest increase in β-sheet content after UV-B treatment (with a *P* value of <0.01 compared to all other ratios). The results also indicated that α-crystallin inhibited β-sheet formation in a concentration dependent manner; a result that was expected and correlated with the molecular function of α-crystallin illustrating our system has similar characteristics as expected for the *in vivo* system. As a molecular chaperone, α-crystallin is the major protein responsible from the lens tissue maintenance by interacting with partially folded proteins to prevent their misfolding and mutual association [53, 54]. It’s also known to inhibit aggregation of various proteins denatured by heat, pH or UV-radiation [15, 55, 56].

**Fig 3:**
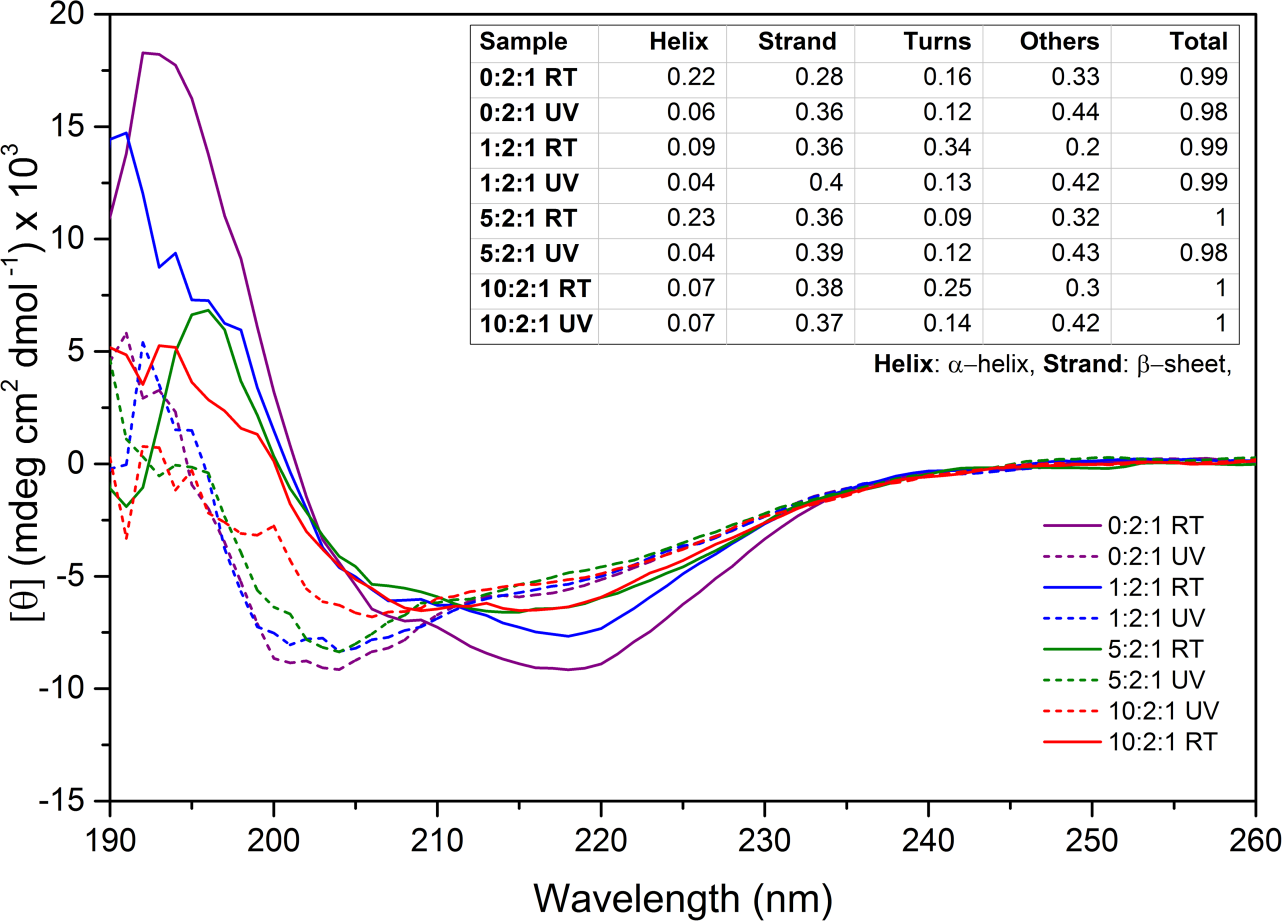
Far UV-CD analysis of crystallin mixture of different ratios (0:2:1, 1:2:1, 5:2:1 and 10:2:1) incubated either at room temperature (solid lines) or under UV-B radiation (dashed lines) for 10 hours. **Inset graph:** The secondary structure predictions acquired with DichroWeb [38, 39] by using CDSSTR [40–42] algorithm. The data fits with an NRMSD value >0.1 were considered as quality fits and represented in the table. (**Helix:** α-helix, **Strand:** β-sheet).

**Fig 4:**
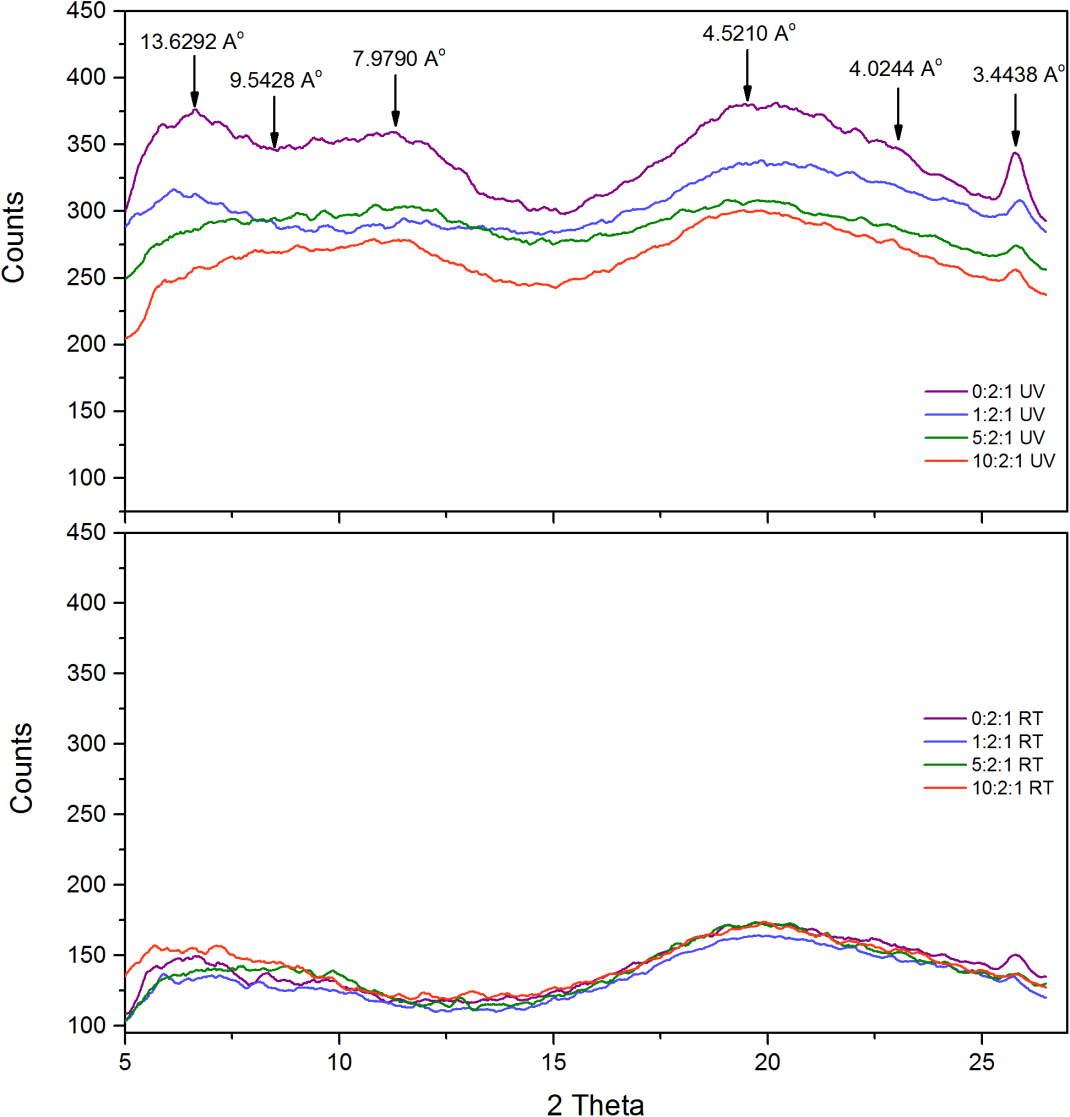
X-ray diffraction analysis of crystallin mixture of different ratios (0:2:1, 1:2:1, 5:2:1 and 10:2:1) incubated either at room temperature (lower graph) or under UV-B radiation (upper graph) for 10 hours.

Following the confirmation of β-sheet structure in UV-B treated samples by using the ThT dye incorporation; the detailed structural changes due to UV-B radiation were studied with far UV-CD (Fig 3) and X-ray diffraction (Fig 4) measurements.

Crystallin proteins gave a CD spectrum indicative of a mixture of α-helix and β-sheet structures (Fig 3, solid lines). There were two minima for all of the samples; the stronger at 208nm and a much weaker one at 218nm representing α-helix and β-sheet structures, respectively [57]. The ratio of these secondary structures with respect to each other was analyzed by using CDSSTR [40–42] algorithm (The fits can be seen in S2 Fig). The analysis showed that α-helix and β-sheet structures comprise 50% of total secondary structures and are approximately in 1:1 ratio for sample 0:2:1 (Fig 3; Inset Table; Helix + Strand). While the overall α-helix/β-sheet structure remained around 50% for all crystallin mixtures; the ratio between α-helix and β-sheet was shifted to the favor of β-sheet with the presence of α-crystallin.

A different spectrum was observed for UV-B treated samples (Fig 3, dashed lines). All of the samples showed a strong minima at ~200nm, which indicates a shift towards random coil structure [57]. Conversely, the broad peak around 208-220nm indicated the contribution of α-helix and β-sheet structures. It is possible that aggregation induced the development of irregular structures during the rearrangement of β-sheets. The calculated secondary structure analysis confirmed that the overall secondary structure was still consisted of ~50% α-helix/β-sheet but overall structure was shifted to random coil. Moreover, the ratio between α-helix and β-sheet contents indicated an increase in β-sheet ratio compared to control (RT) samples. The increase in β-sheet structure was the highest for 0:2:1 and gradually decreased with respect to increasing β-crystallin concentrations. The correlated decrease in α-helix structure suggests that the random coils may arise from the structural change of the α-helix and turn contents of the protein during the β-sheet stacking.

**Fig 5:**
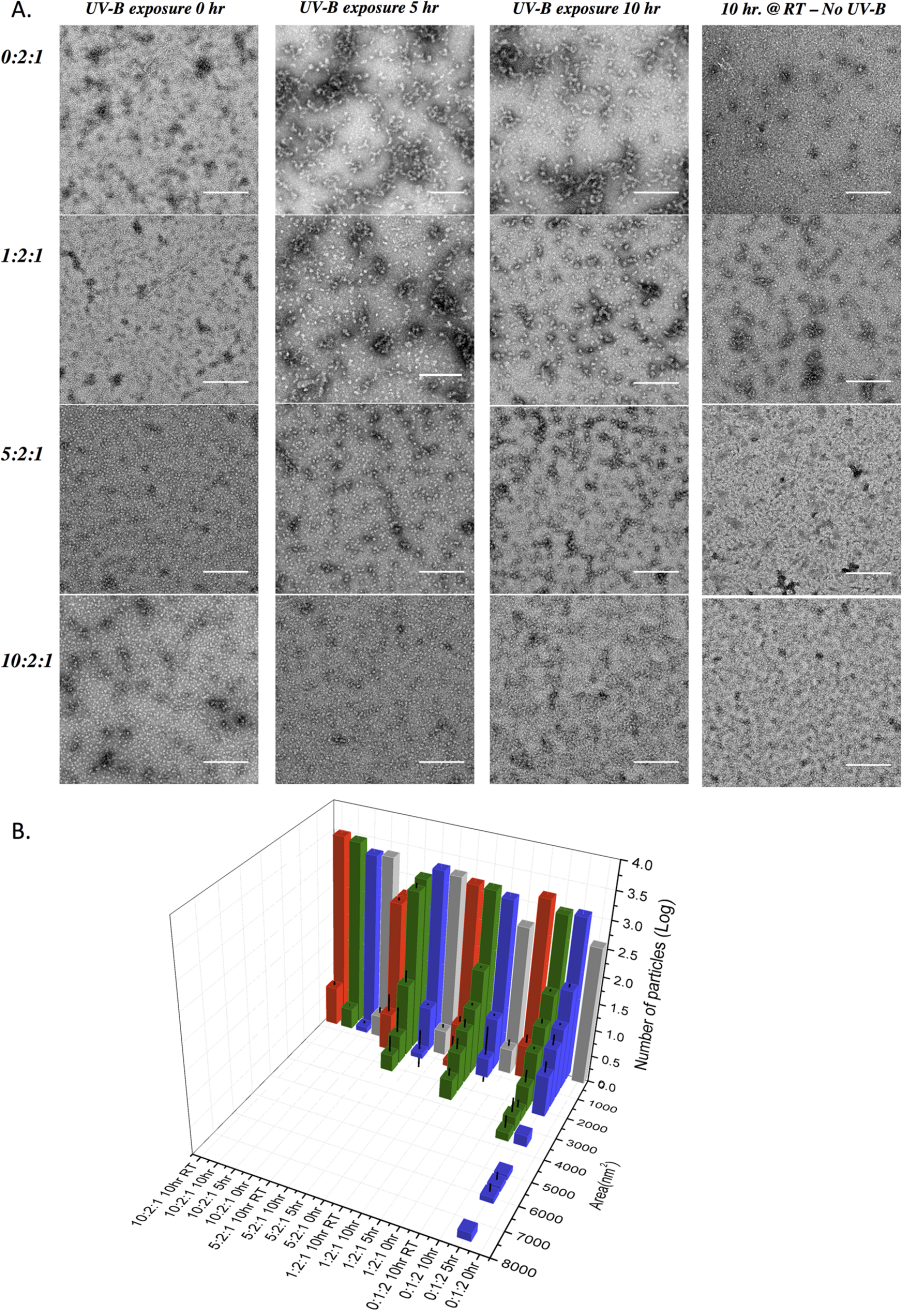
**(A)** Electron micrographs of crystallin fibrils at different ratios (from top to bottom; 0:2:1, 1:2:1, 5:2:1 and 10:2:1) and different times of aggregation (from left to right; 0, 5 and 10 hours of UV-B exposure and 10 hours RT control). **(B)** Surface area distribution of fibrils in each sample. The bars represent the average of at least three images per representative samples. Error bars represent SD.

X-ray diffraction pattern of UV-B treated crystallins proved the presence of β-sheet structure and inter-β-sheet spacing (Fig 4). The indications of cross-β pattern, a sharp 4.7Å meridional reflection (corresponds to the distance between chains in the H-bonding direction) and a broad reflection centred at 9Å on the equator (distance between face-to-face separation of β-sheets), were observed [58]. 7.9Å may represent the interchain helical distances [59]. However, since it was specific for UV-B treated samples, it’s possible that 7.9Å represents the distance between the ends of 2 strands in different sheets during the turn. That explains its dominance in UV-B treated samples since the untreated sample doesn’t contain that organized β-sheet stacking. The peak at 9.6Å is suggestive of an anti-parallel arrangement of β-strands because this spacing would correspond to every other β-strand. Meanwhile, 3.4Å is the distance between α-carbon atoms in anti-parallel β-strands [60]. Together with 3.4Å and 9.6Å peaks, the presence of 13.6Å might be the certain indication of anti-parallel β-strands, where 13.6Å represents the fibril height (radius) composed of 4 amino acids (4 x 3.4Å = 13.6Å). Additionally, the absence of 13.6Å peak in 5:2:1 and 10:2:1 ratios can support the finding of smaller aggregates in these samples (Fig 5).

### Size distribution of UV-B induced fibrils

While SDS-PAGE analysis (Fig 1) confirmed the presence of HMW aggregates in each sample, the β-sheet content were significantly different (Fig 2). This might be due to the different size and shape of the aggregates, which are too large to enter the stacking gel [61]. In order to investigate the possible shape and size variations in samples, TEM (Fig 5) analysis was performed.

No fibrillar or aggregated structures were found before UV-B exposure or after 10 hours incubation at room temperature without UV-B exposure. The images for 0:2:1 and 1:2:1 ratios show the development of fibrillar aggregates after 5 hr UV incubation, which were stable for the following 5 hours (the 10 hr images for 0:2:1 and 1:2:1 ratios). The fibril surface area measurement analysis of the sample 0:2:1, however, indicated the depletion of very large fibrils during the last 5 hours of incubation. This may be the result of amyloid-fibril stabilization and could indicate that the protofibrils don’t interact with each other to form bigger and stable fibrils [2, 48]. On the other hand, 0:2:1 ratio sample contains more fibrillar structures compared to 1:2:1 sample, both at 5hr and 10hr incubation. The presence of α-crystallin should have prevented protein misfolding to some extent, resulting with the inhibition of further fibril growth. That effect of α-crystallin became more evident when analyzing the 5:2:1 and 10:2:1 ratios. Both of the samples indicated the development of aggregates at the end of 5^th^ hour of UV-B exposure and both of them maturated into bigger structures by the end of 10^th^ hour. However none of these structures reach to the size of fibrils obtained from 0:2:1 and 1:2:1 ratios. Moreover, 10:2:1 ratio sample, probably due to the very high concentration of α-crystallin (and relatively decreased concentration of β/γ crystallins) almost completely eliminated large size aggregate formation even after 10 hours of UV-B exposure. Remarkably, neither 5:2:1 nor 10:2:1 ratio samples resulted with fibrillar structures but globular aggregates. Therefore, it is reasonable to speculate that their presence in lens tissue might not to lead cataract formation.

### Alterations in chaperone activity due to UV-B fibrillization

**Fig 6:**
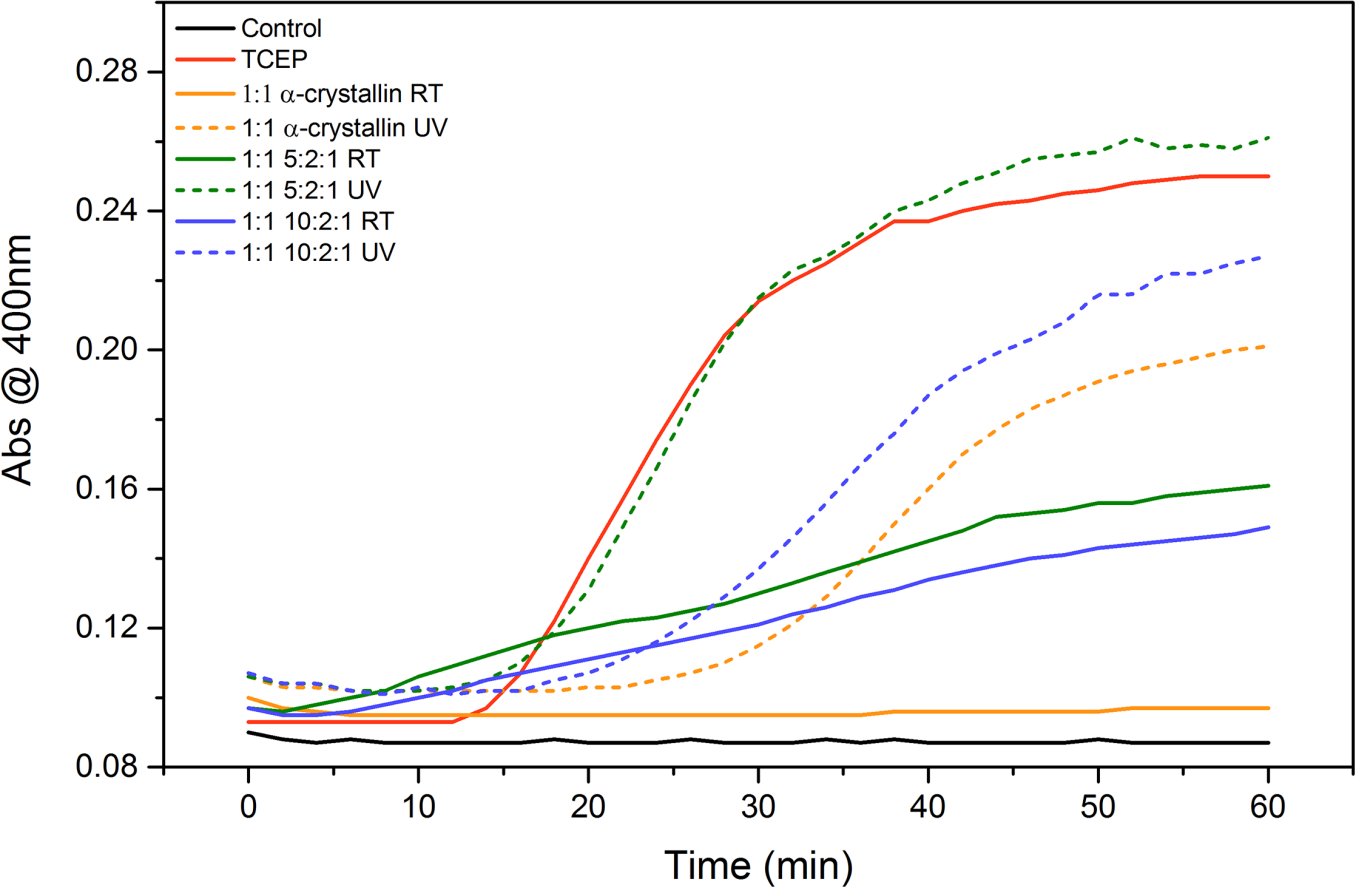
TCEP induced lysozyme aggregation in the absence (red line) and the presence of crystallin proteins (α-crystallin, 5:2:1 and 10:2:1 ratios of α:β:γ crystallins) incubated either at room temperature (solid lines) or under UV-B radiation (dashed lines) for 10 hours. Black solid line represents the native Lysozyme without TCEP addition as control.

As a molecular chaperone, α-crystallin plays a key role in maintaining tissue by inhibiting the fibrillation of crystallin proteins [53]. That function of α-crystallin is not limited with crystallin proteins. It is shown that α-crystallin prevents the aggregation of various proteins denatured by heat, pH or UV-radiation [15, 55, 56, 62]. Thus, the activity of the protein can be measured with a variety of target proteins and denaturing conditions. Here, α-crystallin activity was monitored as the inhibition of TCEP induced lysozyme aggregation. In the presence of TCEP, lysozyme is being denatured and the aggregation is characterized through the increase in turbidity (measured at 400 nm; Fig 6, red line) over a period of 1 hour. Meanwhile, non-denatured lysozyme does not show any aggregation (Fig 6, solid black line).

The positive control of the chaperone activity test was native α-crystallin, which was shown to protect lysozyme from TCEP denaturation for 1 hour (Fig 6, black line). Even after the exposure to UV-B for 10 hours, α-crystallin still retained activity and decreased turbidity (compare Fig 6 orange solid line with orange dashed line). Initially, the 5:2:1 and 10:2:1 samples not exposed to UV-B also provided protection against lysozyme precipitation where more α-crystallin resulted in greater protection. On the other hand, adding the 5:2:1 UV-B treated mixture did not inhibit lysozyme aggregation, indicating the absence of active α-crystallin in the system. The two possible reasons behind that result might be the inactivation of all the α-crystallin due to UV-B exposure or the incorporation of all the α-crystallin into the aggregates regardless of denaturation. Similar results were obtained for 1:2:1 ratio indicating the absence of free and/or active α-crystallins in the system (data not shown). However, 10:2:1 mixture retained α-crystallin activity after UV-B exposure. It’s possible that there is free α-crystallin, which didn’t become a part of the aggregates only at 10:2:1 ratio.

## Conclusion

Cataract is considered as a protein aggregation disease. The natural pathways of ageing (oxidative stress) and exposure to sunlight induce the oxidation, cross-linking, cleavage, and deamination of crystallins resulting in the aggregation of proteins and eventually the formation of cataract. Many denaturant conditions can provoke aggregation of crystallin proteins *in vitro*. UV-B radiation can mimic the natural system and provides an appropriate platform to investigate cataractous fibrils and possible treatment strategies.

Here, we investigated the fibrillation of α:β:γ crystallin protein mixtures. It was possible to induce fibrillation of physiologically relevant ratios of crystallin proteins with UV-B radiation. Moreover, time-dependent fibrillation profile was found to be similar with the gradual formation of age-related nuclear cataracts [45]. Formed fibrils were inhibited by the presence of the molecular chaperone of the eye, α-crystallin, as it would be in natural tissue. Furthermore, our results confirmed the development of amyloid-like fibrils, which are in cross-β-sheet form and possibly in anti-parallel β-sheet arrangements.

In light of our findings, we conclude that UV-B induced fibrils of α:β:γ crystallin mixtures can serve as a platform for studying cataract formation, which may also enable the testing of alternate therapies for treatment (e.g. drug candidates). We envision that in the follow-up studies utilization of higher protein concentrations (as high as found in natural lens tissue ~300 mg/ml) and different sub-types of α-, β- and γ-crystallin families would advance this system to a more valid platform. We also believe that further structural analysis of these fibrils using techniques like solid-state NMR would provide insight about the nature of the cataractous fibrils.

## Supporting information

S1 File Expression and purification of Human crystallin proteins. (DOCX)

S1 Fig The color of crystallin mixture ratio 1:2:1 before and after UV-B radiation for 10 hr. (DOCX)

S2 Fig CD spectrums and resulting fits. (DOCX)
