# Supplementary material for: UV-B induced fibrillization of crystallin protein mixtures
Source: PLoS One. 2017 May 25;12(5):e0177991. doi: 10.1371/journal.pone.0177991 (PMC5444657; doi:10.1371/journal.pone.0177991)
Supplement: S1 File — (DOCX) [file pone.0177991.s001.docx]

**S1 File: Expression and purification of Human crystallin proteins**

**Protocol A: Expression and purification of Human crystallin proteins**

Recombinant proteins were expressed in *E. coli BL21(DE3)* host cells in LB medium supplemented with 100 μg/mL ampicillin. The cultures for the human crystallin βB2 and γD were grown at 37 °C while αB construct were grown at 20 ^o^C until an OD_600_ = 0.6 was reached and the cells were induced with 1.0 mM IPTG for 18 hr at 37°C. After induction the cells were harvested by centrifugation at 6076 x g for 20 min at 4 °C.

Recombinant human crystallin αB, βB2 and γD were purified in the same manner using metal affinity chromatography (IMAC) with Ni-NTA column and enterokinase digest for the next N-terminal His-tag removal.

The cell pellets from 1 L of the LB medium with expressed crystallin protein were re-suspended in 25 mL of buffer A (Buffer A: 25 mM Tris, protein inhibitor EDTA-free tablet, pH 8.0) and lysed by sonication (using Fisher Scientific Ultrasonic Dismembrator Model 500 with microtip probe for 4 X15sec pulses at 45% power) on ice followed by ultracentrifugation at 223000 x g (55000 rpm using 70 Ti rotor from Beckman coulter) for 120 min at 10 °C. The supernatant was removed and loaded onto the Ni-NTA column (12.5 mL bed volume) pre-equilibrated with buffer A. The column was then washed with buffer B, buffer C and buffer D sequentially (Buffer B: 25 mM Tris, 0.1% Triton X-100, pH 8.0; Buffer C: 25 mM Tris, 1M NaCl, pH 8.0; Buffer D: 25 mM Tris, 25mM Imidazole, pH 8.0) and crystalline protein was eluted with a linear gradient of 500 mM imidazole in buffer A. Eluted fractions were subjected to the SDS-PAGE electrophoresis (12% Tris-Glycine gel). Fractions with identified crystallin were mixed followed by dialysis at 4^o^C with two buffer changes against 10 volumes of buffer A for the imidazole removal. After dialysis was completed, CaCl_2_ was added to the 2mM final concentration.

Crystallin protein was mixed with enterokinase (0.02μg of the enterokinase per 1mg of the crystalline) and incubated for 22 hr. at room temperature for His-tag removal. His-tag digestion was monitored by SDS-PAGE analysis (Fig A). Crystallin sample was passed through Ni-NTA resin and trypsin inhibitor agarose for the His-tag and enterokinase removal. To inhibit all possible traces of the enterokinase activity PMSF was added to the protein with 0.1mM final concentration. The protein concentration was determined using the corresponding extinction coefficient at 280 nm (crystallin αB: 13980 M^−1^ cm^−1^, crystallin βB2: 41035 M^−1^ cm^−1^ and crystallin γD: 43235 M^−1^ cm^−1^) calculated by the Protparam software based on recombinant crystalline protein sequences. All proteins were finally concentrated using an Amicon Ultra-15 centrifugal device. In order to confirm the protein sequences, each protein run in SDS-PAGE gel were cut and subjected to In Gel Protein Identification analyses performed at Alberta Proteomics and Mass Spectrometry Facility - University of Alberta (Fig B).

**Protocol B: SDS-PAGE analysis**

Proteins were analyzed using 15% standard Laemmli SDS-PAGE electrophoresis with 10% β-mercaptoethanol freshly added to the loading buffer. Invitrogen™ Novex™ XCell™ SureLock™ mini gel cell was operated under constant 200 V for 57 min. Separated protein bands were stained with Coomassie Brilliant Blue staining. PageRuler™ Prestained Protein Ladder (ThermoFisher Scientific) was used as a reference.

**Fig A.** Schematic representation of pET15 b vector constructs for crystallin proteins **(A)**. SDS-PAGE images for; αB crystallin purification with increasing Imidazole concentration **(B)**, pure αB crystallin **(C. Lane 1)**, enterokinase digested αB crystallin **(C Lane 2)**, βB2 crystallin purification with increasing Imidazole concentration **(D)**, pure βB2 crystallin (E Lane 1), pure enterokinase digested βB2 crystallin **(E Lane 2)**, ΥD crystallin purification with increasing Imidazole concentration **(F)**, pure ΥD crystallin **(G Lane 1)**, enterokinase digested ΥD crystallin **(G Lane 2)**, pure enterokinase digested ΥD crystallin **(G Lane 3)**.

**Fig B.** Protein identification results for recombinant αB, βB2 and γD human-crystallin proteins. **Accession;** The unique protein assention number for the identification. **Description;** Protein name. **Score;** Sequest score. **Coverage;** Percent coverage of the protein observed. **# Proteins;** Number of proteins matching the peptides identified. **# Unique Peptides;** Unique peptides identified which only occur in the protein identified**. # Peptides;** All unique peptide plus peptides that may be common between two or more proteins. **#PSMs;** Peptide Spectral Matches. PSMs are roughly proportional to protein abundance.
