# Supplementary material for: UV-B induced fibrillization of crystallin protein mixtures
Source: PLoS One. 2017 May 25;12(5):e0177991. doi: 10.1371/journal.pone.0177991 (PMC5444657; doi:10.1371/journal.pone.0177991)
Supplement: S1 Fig — (DOCX) [file pone.0177991.s002.docx]

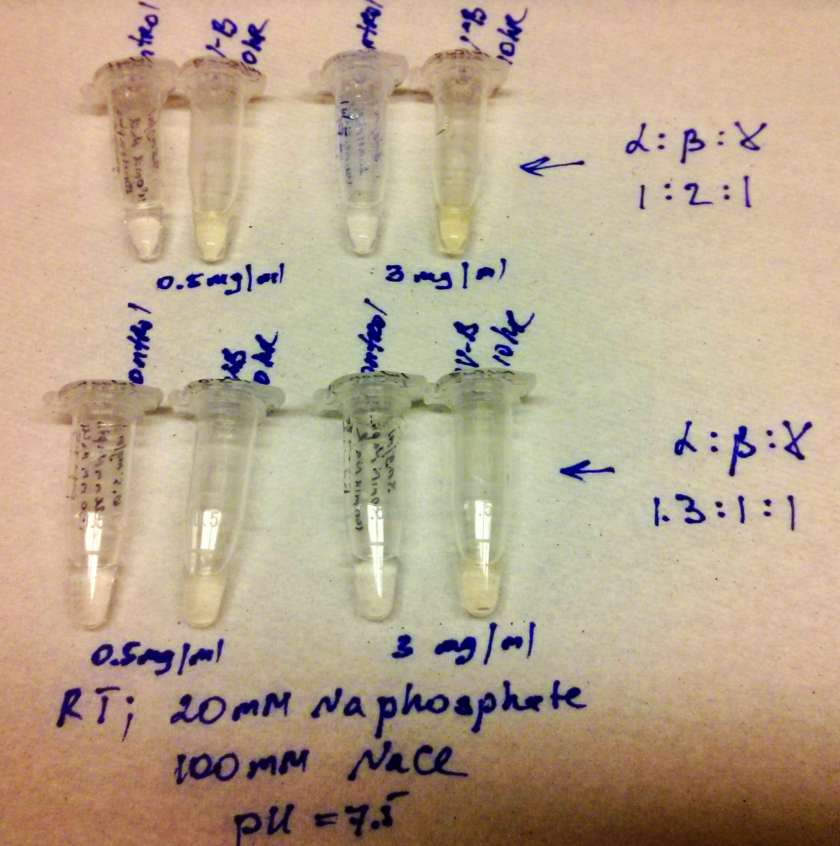


**S1 Fig.** The color of crystallin mixture ratio 1:2:1 before and after UV-B radiation for 10 hr. The concentration of the protein mixture is reflected in the color intensity.
