# Supplementary figures and images for: UV-B induced fibrillization of crystallin protein mixtures
Source: PLoS One. 2017 May 25;12(5):e0177991. doi: 10.1371/journal.pone.0177991 (PMC5444657; doi:10.1371/journal.pone.0177991)

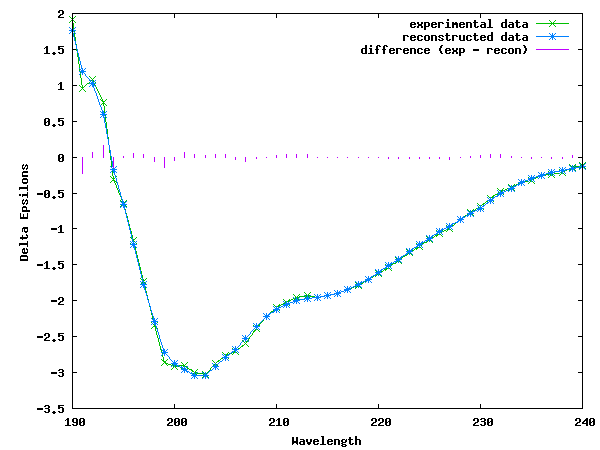

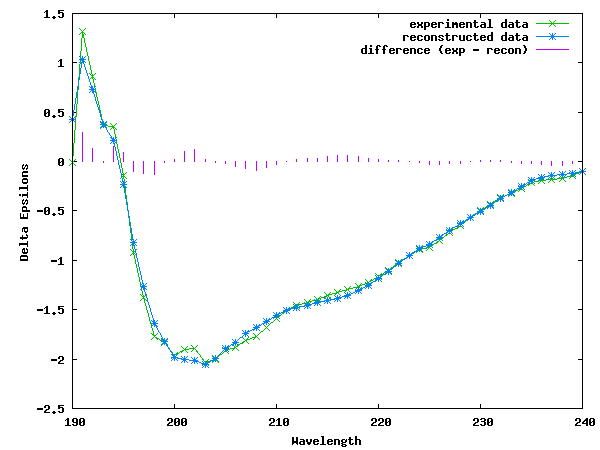

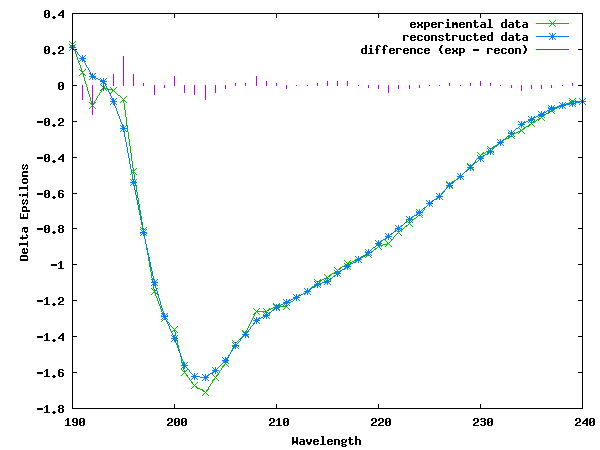

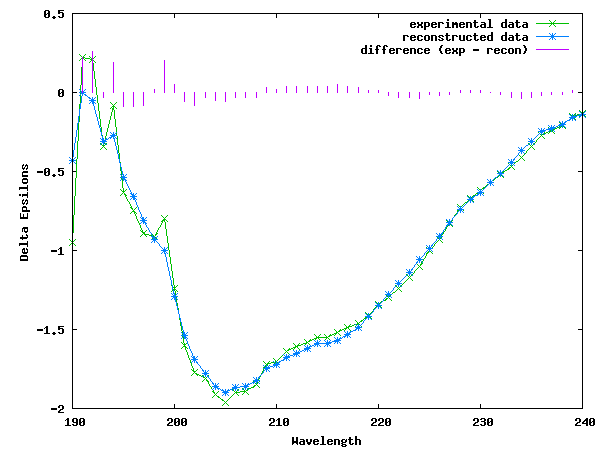

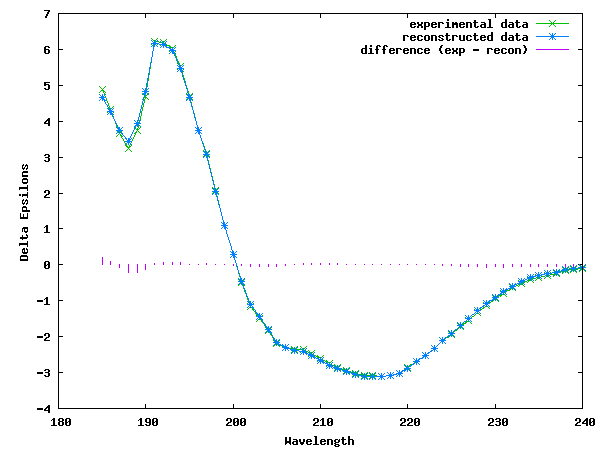

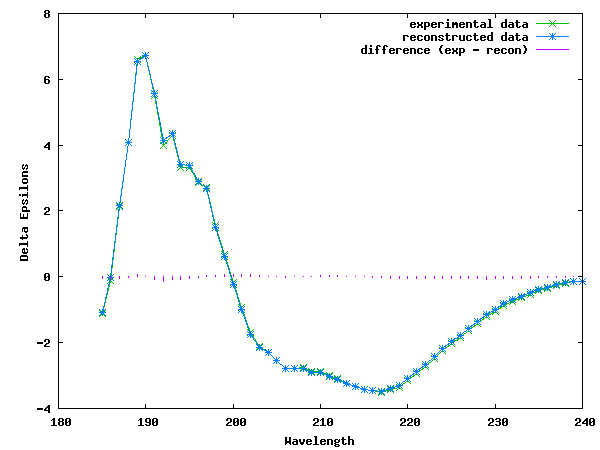

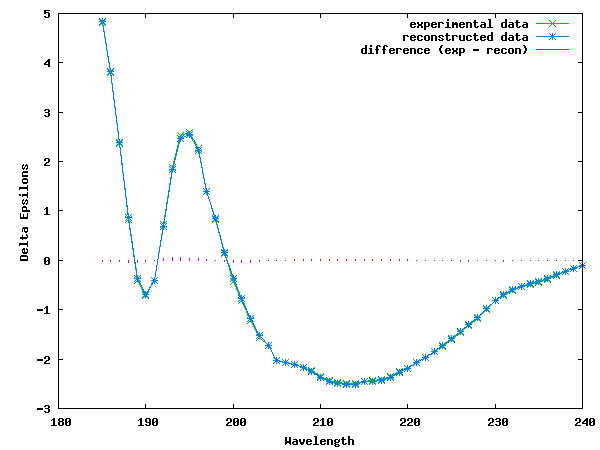

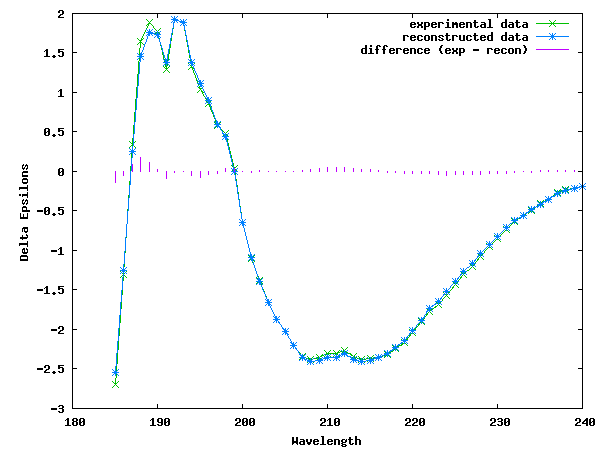


**A.**

**B.**

**C.**

**D.**

**E.**

**F.**

**G.**

**H.**

**S2 Fig.** CD spectrum and resulting fit with CDSSTR for **A;** 0:2:1 RT, **B;** 0:2:1UV, **C;** 1:2:1RT, **D;** 1:2:1UV, **E;** 5:2:1RT, **F;** 5:2:1UV, **G;** 10:2:1RT, **H;** 10:2:1UV

Supplement: S2 Fig — (DOCX) [file pone.0177991.s003.docx]
